# Harnessing Eugenol for Sustainability: Synthesis and Characterization of Biobased Epoxy Thermosets

**DOI:** 10.1021/acsomega.5c10201

**Published:** 2026-03-09

**Authors:** Mădălina I. Necolau, Brînduşa Bălănucă, Celina M. Damian, Horia Iovu

**Affiliations:** 1 Advanced Polymer Materials Group, National University of Science and Technology POLITEHNICA, Bucharest 011061, Romania; 2 Faculty of Chemical Engineering and Biotechnology, Department of Organic Chemistry “C. Nenitescu”, National University of Science and Technology POLITEHNICA, Bucharest 011061, Romania; 3 Academy of Romanian Scientists, Ilfov 3, Bucharest 050044, Romania

## Abstract

In this study, biobased epoxy networks were developed from a bis-eugenol epoxy monomer (B) cured with natural carboxylic acids such as citric acid and itaconic acid (IA) and with synthetic amine Jeffamine D230 (D). Differential scanning calorimetry revealed distinct curing behaviors governed by the functionality and structure of the cross-linkers. The IA-cured network showed dual reactivity through its α,β-unsaturated double bond, leading to additional cross-linking at elevated temperatures. Fourier transform infrared spectroscopy confirmed complete epoxy conversion via esterification or amino-addition mechanisms, while thermogravimetric analysis demonstrated high char yields and no solvent residues, indicating excellent thermal stability and potential flame-retardant behavior. Dynamic mechanical analysis revealed glass transition temperatures of 48–56 °C and gel contents above 96%, confirming full curing and dense cross-linked structures. Surface analyses showed tunable hydrophilicity and improved antibacterial activity for acid-cured systems. Degradation and XPS studies further highlighted that the chemical nature of the cross-linker dictates stability under corrosive environments. Amine-cured networks degrade via C–O cleavage in aqueous and alkaline media, whereas acid-cured systems resist oxidation but undergo ester hydrolysis under basic conditions. These findings demonstrate that eugenol-based thermosets can serve as eco-friendly, corrosion-resistant coatings with adjustable flexibility and high renewable content.

## Graphical abstract

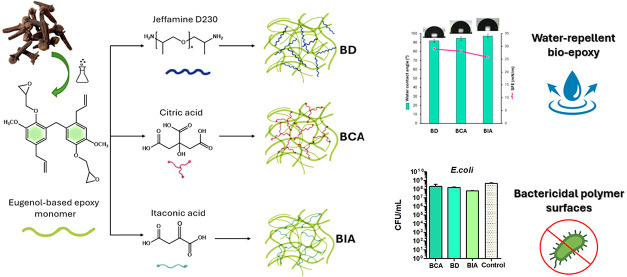

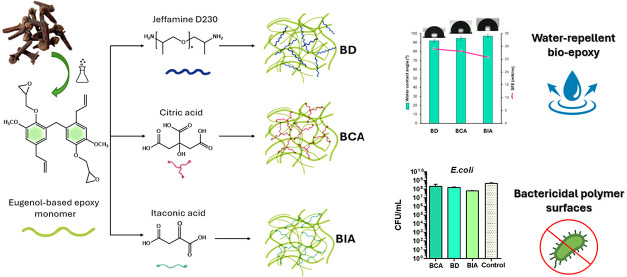

## 1 Introduction

Epoxy resin (ER) is one of the most important thermosetting materials, with broad industrial applicability in protective coatings, structural adhesives, and composite formulations. This versatility is primarily attributed to its superior mechanical performance, excellent chemical resistance and adhesion, and high thermal stability.^1–3^

The growing demand for advanced materials, coupled with increasing environmental concerns, has stimulated the development of sustainable epoxy systems as eco-friendly alternatives to conventional petroleum-based thermosets.^4–6^ In addition to the excessive exploitation of nonrenewable resources, the extensive use of bisphenol A, an essential precursor in epoxy resin synthesis, represents a major concern for industry. Bisphenol A is classified as an endocrine-disrupting chemical and exhibits significant toxicity.^7–9^ Due to its structural similarity to estrogen, even small amounts can lead to hormonal imbalances, reproductive disorders, and potentially cancer.^10–12^

Moreover, epoxy coatings can gradually release trace amounts of bisphenol A through incomplete polymerization, hydrolysis, or environmental degradation.^13^ Brunchet et al. demonstrated that epoxy coatings used to protect pipes and tanks undergo moderate oxidation in chlorinated tap water.^14^

In this context, replacing bisphenol A with green or biobased counterparts that possess similar structural features is considered a promising approach. Several studies have reported the synthesis of biobased ERs derived from biomass, including vegetable oils, such as soybean, linseed, castor, cottonseed, camelina, and karanja oil, whose long-chain structures typically lead to highly flexible materials.^15–17^

However, considering both the drawbacks of bisphenol A and the structural advantages it imparts, particularly its rigid phenolic architecture, it is worth exploring naturally occurring phenolic compounds (e.g., vanillin, guaiacol, and eugenol) as potential alternatives.^18–20^

Eugenol (4-allyl-2-methoxyphenol), the main component of clove oil (about 80%),^21^ is a naturally occurring phenolic compound with two key functional groups: a terminal vinyl group and a hydroxyl group. These functionalities offer multiple possibilities for chemical modification, enabling the synthesis of a wide variety of derivatives.^22^ Due to its rigid aromatic ring and methoxy substituent, polymers derived from eugenol are expected to exhibit excellent mechanical strength and thermal resistance.^23^ Furthermore, the phenolic hydroxyl group can be converted into a glycidyl ether via reaction with epichlorohydrin, a transformation analogous to that used for bisphenol A. This promising structural similarity has already attracted considerable attention, and several eugenol-based epoxy formulations have been reported.^24–26^

Various types of eugenol-derived thermosetting resins have been reported, including epoxy, benzoxazine, urethane, and acrylate systems. Eugenol-based epoxy resins, obtained through glycidylation or allylation, are typically cured using amines such as 4,4′-diaminodiphenylmethane (DDM), isophorone diamine (IPDA), or anhydride hardeners, resulting in glass transition temperatures (Tg) ranging from 80 to over 200 °C and decomposition temperatures above 300 °C, depending on the cross-link density and curing agent.^23,27–31^ Eugenol-based benzoxazine resins undergo thermal ring-opening polymerization, yielding materials with excellent thermal stability and char yields exceeding 30% at 800 °C, suitable for high-performance coatings and composites.^32^ Eugenol-urethane systems, synthesized via the reaction of eugenol-based diols or polyols with isocyanates, exhibit tunable flexibility and good thermal resistance, with Tg values between 50 and 130 °C.^33^ Furthermore, eugenol acrylates and methacrylates have been developed for UV-curable coatings and adhesives, where cationic or radical photopolymerization leads to moderate Tg (60–100 °C) and rapid curing kinetics.^34^ Collectively, these studies demonstrate the versatility of eugenol as a renewable feedstock capable of forming thermosets with competitive thermal and mechanical performance compared to conventional bisphenol A-based systems.

Based on the above findings, it can be noted that the biobased eugenol-derived ER still needs further modification to minimize the use of toxic solvents and reagents and also to understand how eugenol building blocks behave during curing reactions. The existing synthesis and purification methods of eugenol-based epoxy monomers still need optimization regarding the preparation steps and separation process. The mechanical and thermal properties of eugenol-based ER require improvement, and the development of diglycidyl ether of bisphenol A (DGEBA) homologues derived from natural sources is imperatively needed.

This work provides the first systematic comparison of eugenol-based epoxy networks cured with three structurally distinct hardeners: an amine, a tricarboxylic acid, and an unsaturated dicarboxylic acid. By correlating their curing mechanisms with thermal behavior, hydrolytic or oxidative degradation pathways, and antibacterial performance, the study delivers an integrated structure–property–function insight that is not available in the current literature. This comprehensive evaluation establishes how the choice of bioderived cross-linker governs the architecture and functionality of eugenol-based thermosets.

## 2 Materials and Methods

### 2.1 Materials

Eugenol from Roth (Mw = 164.21 g/mol), orthophosphoric acid (85.7%) from Fisher Chemical, formaldehyde (37%) from CHIMREACTIV SRL, epichlorohydrin (ECH, 99%, Mw = 92.52 g/mol) from Sigma-Aldrich, tetrabutylammonium bromide (TBAB, Mw = 322.37 g/mol) from Sigma-Aldrich, sodium hydroxide (NaOH, ACS reagent, ≥97.0%), and toluene (anhydrous, 99.8%) from Sigma-Aldrich were used as received.

For the curing process, tetrahydrofuran (THF), citric acid (CA, ACS reagent, ≥99.5%), itaconic acid (IA, ≥99%), and Jeffamine D230 polyetheramine from Huntsman were employed.

### 2.2 Methods

#### 2.2.1 Synthesis of the Eugenol-Based Epoxy Monomer

The synthesis of the eugenol-based monomer followed a two-step protocol adapted from the literature.^35^ In the first stage, the condensation of eugenol molecules was required to obtain a structure closer to the classical DGEBA. For that, eugenol (0.22 mol) was reacted with orthophosphoric acid (7 mL, 85 wt %) in a three-necked flask equipped with a magnetic stirrer for 30 min at 50 °C. Formaldehyde solution (0.1 mol, 38 wt %) was then added dropwise within an hour, and the mixture was further reacted for 6 h at 90 °C under a N_2_ atmosphere. After completing the synthesis, the product was washed with deionized water until neutral pH was reached.

In the next stage of the reaction, 10 g of condensed eugenol was reacted with 36.7 mL of epichlorohydrin in the presence of 0.3 g of tetrabutylammonium bromide for 6 h at 90 °C. The excess of epichlorohydrin was then removed under reduced pressure, and the resulting eugenol-epichlorohydrin prepolymer was solubilized in 36.7 mL of toluene and reacted with 8.47 g of NaOH (50 wt % water solution) for another 3 h at 90 °C. A yellow viscous product was obtained (with a yield of 84%), which was washed with deionized water until neutralization. The reactions involved in each step are depicted in Figure 1.

**Figure 1:**
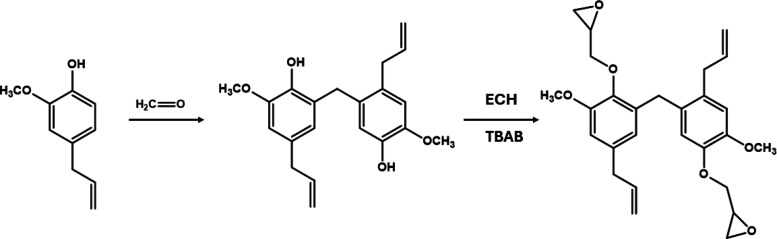
Schematic representation of the main reactions involved in the synthesis of the diepoxide eugenol-based monomer.

#### 2.2.2 Cross-Linking Process for the Biobased Epoxy Monomer

The obtained diepoxide eugenol-based monomer (B) was further cross-linked by employing different polymerization mechanisms. Carboxylic acids of natural origin used for the curing process were citric acid (CA) and itaconic acid (IA), and the stoichiometry was calculated based on the number of reactive groups, maintaining an equivalent ratio of carboxylic-to-epoxy functionalities (COOH:epoxy = 1:1 eq). Jeffamine D230 (D), the conventional primary amine required to cure the bioepoxy monomer, was determined considering the amine index and was also stoichiometrically considered (active H:epoxy = 1:1). Prior to polymerization, the carboxylic acids were solubilized in the minimal volume of THF necessary to achieve complete dissolution at 50 °C and the as obtained solution was added to the epoxy monomer and homogenized. The reaction mixtures were subsequently maintained at 60 °C for 10 min under continuous stirring in an open system to allow complete evaporation of THF, ensuring solvent-free prepolymers before curing.

The curing temperatures were selected based on preliminary differential scanning calorimetry (DSC) analyses, which identified the onset and peak of the curing exotherms for each epoxy/curing-agent system. The thermal schedules were optimized to ensure complete cross-linking of all formulations, and they are presented in Table 1.

**Table 1:** Sample Abbreviations and Curing Parameters for the Synthesized Biobased Epoxies

Nr.crt	cross-linking agent	sample abbreviation	temperature for the curing (°C)
1	Jeffamine D230	**BD**	50
			80
2	citric acid	**BCA**	100
			120
3	itaconic acid	**BIA**	100
			150

The possible mechanisms of the curing process for eugenol-based epoxy monomer with the selected cross-linking agents is depicted in Figure 2.

**Figure 2:**
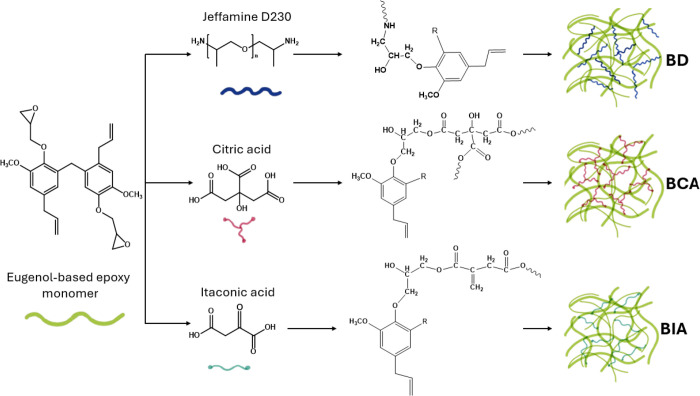
Possible reaction pathway for the eugenol-based epoxy monomer ring-opening reactions in the presence of Jeffamine D230 (BD), citric acid (BCA), and itaconic acid (BIA) as cross-linking agents.

### 2.3 Characterization

#### 2.3.1 Fourier Transform Infrared Spectroscopy (FTIR)

FTIR was employed to confirm the chemical structure of the biobased epoxy monomer, and the results were recorded on Bruker Vertex 70 equipment in the 400–4000 cm^–1^ range with 4 cm^–1^ resolution and 32 scans. The samples were analyzed on an attenuated total reflection (ATR) module.

#### 2.3.2 ^1^H Nuclear Magnetic Resonance (^1^H NMR)

^1^H NMR spectra were recorded on a Bruker Advance III HD 600 MHz spectrometer using CDCl_3_ as a solvent.

#### 2.3.3 X-ray Photoelectron Spectrometry (XPS)

XPS analysis was conducted on a K-Alpha spectrometer with a monochromatic Al Kα source (1486.6 eV) working at a vacuum base pressure of 2 × 10^–9^ mbar. Charging effects were compensated using a flood gun, and binding energy was calibrated by placing the C1s peak at 284.8 eV as an internal standard. Deconvolution of C 1 s peaks was performed by using a smart background algorithm with a convolved Gaussian–Lorentzian ratio. The pass energy for the survey spectra was set at 200 eV, while for the high-resolution C1 s spectra acquisition, it was 20 eV.

#### 2.3.4 Epoxy Equivalent Weight (EEW)

EEW is an essential parameter based on which the proper amount of curing agent can be established. The EEW is defined as the weight in grams of an epoxy resin containing one mole equivalent of epoxide (g/mol).^36^ The epoxy equivalent of the newly synthesized eugenol-based epoxy resin (ECE) was determined using the hydrochloric acid-acetone titration method^37^ with the help of the following equation:

$$\mathrm{EEW}=\frac{m\times 1000}{\left(V_{0}-V_{1}\right)\times c\times F}$$ 1

where *m* is the quantity of epoxydic resin subjected to analysis, g; *V*_0_ is the volume of KOH 0.1 N solution used for the titration of the blank sample, mL; *V*_1_ is the volume of KOH 0.1 N solution used for the titration of the sample containing epoxy resin, mL; *c* is the concentration of KOH solution, N; and *F* is the volumetric factor for the KOH 0.1 N solution. Following this method, the EEW for the eugenol-based epoxy monomer was found to be 178 eq epoxy/g.

#### 2.3.5 Gel Content (GC)

GC was determined by placing around 0.5 g of each sample in THF for 48 h. After that, the samples were dried at 70 °C for 24 h and the gel content was calculated with the aid of the following equation:

$$\mathrm{GC}=\frac{m_{2}}{m_{1}}\cdot 100$$ 2

where *m*_2_ is the mass of the dried sample and *m*_1_ is the initial mass of the sample.

#### 2.3.6 Differential Scanning Calorimetry (DSC)

DSC was performed on a Netzsch DSC 204 F1Phoenix equipment. The samples after the prepolymerization step were subjected to heating from room temperature (RT) to 300 °C with a rate of 5 °C/min under nitrogen (20 mL/min flow rate). The curing conversion was monitored through nonisothermal analysis of samples collected at specific times of reaction while maintained in a heated oven. The temperatures employed for the kinetic study of each system are depicted in Table 1. Curing conversion (α) was calculated based on eq 3 from the evolution of Δ*H* with temperature at different reaction times.

$$\alpha =\frac{\Delta H_{0}-\Delta H_{t}}{\Delta H_{0}}\cdot 100=\frac{\Delta H_{\mathrm{rez}}}{\Delta H_{0}}$$ 3

where α is the conversion of epoxy functional groups, %; Δ*H*_0_ is the total enthalpy of reaction at the moment *t* = 0, J/g; and Δ*H*_*t*_ is the enthalpy of the reaction at the moment *t*, J/g.

The apparent activation energy (Ea) was calculated based on the DSC kinetic results by using the logarithmic form of the Arrhenius equation:

$$\ln K=\ln A-\frac{\mathrm{Ea}}{RT}$$ 4

where *K* represents the rate constant (determined from the slope of the kinetic curves), *A* is the pre-exponential factor, Ea is the activation energy, *R* is the universal gas constant, and *T* is the temperature in K.

#### 2.3.7 Dynamic Mechanical Analysis (DMA)

DMA was performed on a TRITEC 2000 B equipment in single cantilever bending mode at 1 Hz frequency in the temperature range of −80 to 120 °C with a heating rate of 4 °C/min. The cross-linking density (υe) of the synthesized bioepoxy networks was calculated from DMA results based on the theory of rubber elasticity using the following equation:^38^

$$\upsilon e=G^{\prime }/3TR$$ 5

where *G*′ is the storage modulus in the rubbery plateau region (MPa), *T* is the temperature in kelvin, and *R* is the universal gas constant (8.314 MPa cm^3^/mol K).

#### 2.3.8 Thermogravimetric Analysis (TGA)

TGA was performed using Netzsch TG 209 F1 Libra equipment, from RT to 800 °C under a nitrogen atmosphere with a heating rate of 10 °C/min. Residual mass from TGA results was used to determine the limiting oxygen index (LOI) using Van Krevelen’s empirical eq 6.^39^ Approximately 10 ± 1 mg of each cured sample was analyzed in triplicate.

LOI=17.5+0.4×charyield 6

#### 2.3.9 Contact Angle Measurements

The contact angle measurements were performed using the drop shape analyzer-DSA100 from Krüss Scientific GmbH with water and ethylene glycol as probing liquids. The surface free energy was automatically computed using the Young–Laplace fitting method, which implies the work of adhesion by the Young–Drupe equation.

#### 2.3.10 Antibacterial Tests

The antibacterial tests were performed according to CLSI 2018 M07. *Escherichia coli* (*E. coli*) ATCC 25922 with a density of 10^9^ UFC/mL (colony-forming units) was adjusted according to the mass of the samples. The material samples, previously sterilized by UV exposure (30 min on each side), were placed in contact with the microbial inoculum for 24 h and centrifuged on a vortex. After 24 h, 6 decimal serial dilutions were performed to determine the logarithmic and percentage reduction of the microbial populations. The control used in the antibacterial test was a positive growth control consisting of *E. coli* ATCC 25922 cultured under identical conditions but without any material sample.

Then, 10 μL was used in triplicate, which was inoculated at one spot on solid medium plate count agar. After 18–24 h of incubation at 36 °C, the plates were read by counting colonies.

#### 2.3.11 Material Behavior in Different Corrosive Media

Samples were analyzed for their resistance in different media: water, NaCl solution (3.5%), base solution (NaOH, 1 M), and acid solution (H_2_SO_4_, 1 M), following an adapted procedure described in the ASTM D570 standard. Each test was performed in triplicate. BD, BCA, and BIA were weighed and placed in the incubation medium, and at different times (1, 7, 10, and 14 days), each sample was weighed again, after removing the excess liquid. The results have been expressed as swelling degree (SD), using the equation below:

$$\mathrm{SD}\left(\%\right)=\frac{m_{t}-m_{0}}{m_{0}}\cdot 100$$ 7

where *m*_0_ is the mass of the dried sample and *m*_t_ is the mass of the sample at a specific time.

## 3 Results and Discussion

### 3.1 Structural Characterization

FTIR analysis revealed the characteristic features of the newly synthesized eugenol-based epoxy monomer, as shown in Figure 3. The spectrum for eugenol exhibits characteristic bands for phenolic compounds, including the phenolic hydroxyl group at 3519 cm^–1^ and the skeletal vibrations associated with the aromatic ring at 1513 and 1610 cm^–1^.^40^ In addition, the allylic C=C stretching vibration appears at 1610 cm^–1^ while the linkage between the benzene ring and the methoxy group is observed in the 1100–1200 cm^–1^ region.^41^

**Figure 3:**
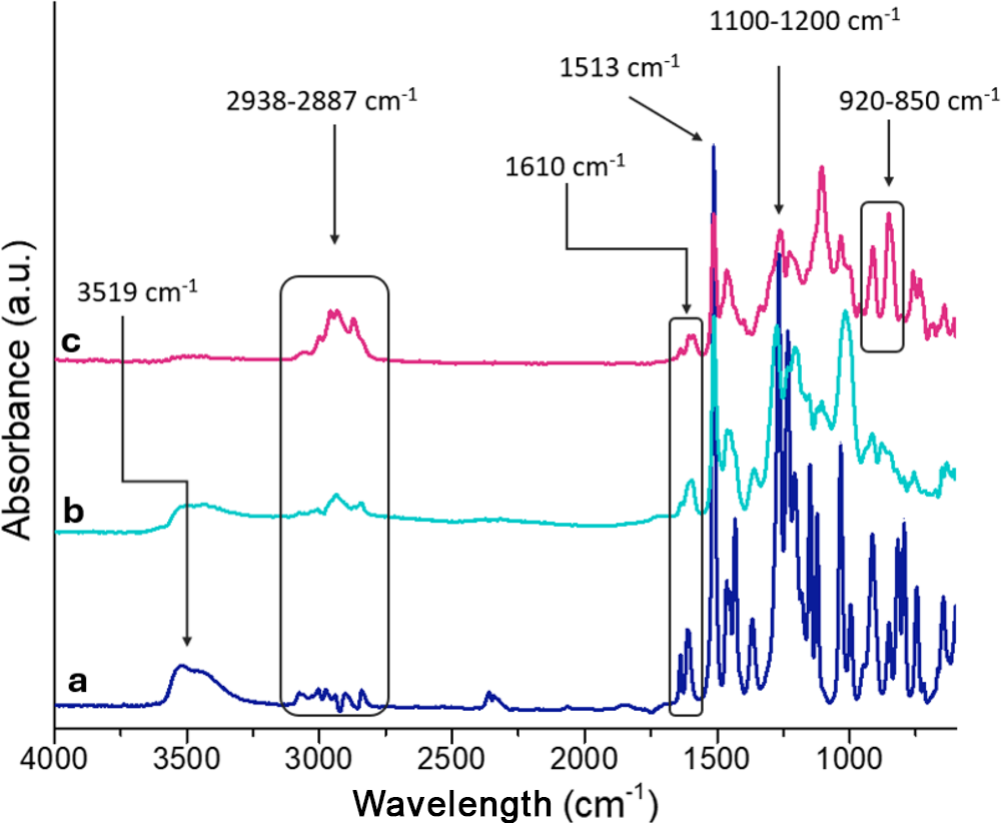
FTIR spectra for (a) eugenol, (b) condensed eugenol, and (c) eugenol-based epoxy monomer.

The spectrum for epoxidized eugenol (Figure 3c) displays the characteristic absorption bands of the oxirane ring with signals appearing at 849 and 915 cm^–1^. In addition, the cluster peaks at 2877 and 2938 cm^–1^ correspond to the CH_2_ stretching vibrations associated with the epoxy group.^42^

Compared with the spectra for eugenol and bis-eugenol, in the epoxidized structure, the absence of the −OH stretching band in the epoxidized derivative indicates that the hydroxyl groups have fully reacted with epichlorohydrin, thereby confirming the successful formation of the epoxy monomer.^43^

^1^H NMR (CDCl_3_, TMS, δ in ppm) structural characteristics are shown in Figure 4 to address the synthesis of a sustainable monomer based on eugenol.

**Figure 4:**
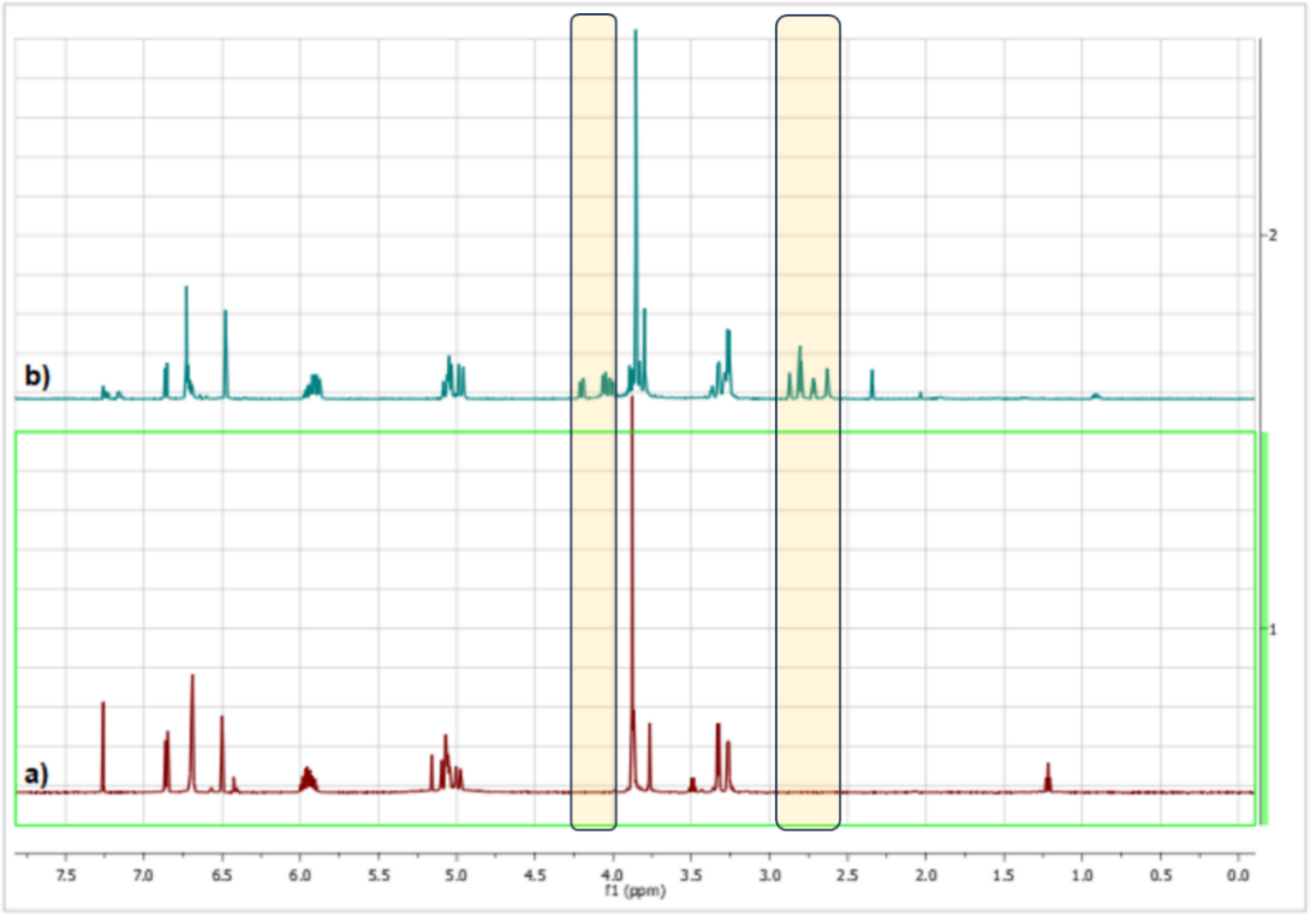
^1^H NMR spectra of (a) eugenol and (b) the epoxy derivative.

Figure 4 presents the ^1^H NMR spectra of the biphenolic derivative of eugenol (a) and the epoxy derivative of eugenol (b). The appearance of the characteristic signals of the epoxide moieties at 2.88–2.64 and 4.21–3.81 ppm confirms the successful grafting of the glycidyl groups onto the condensation product. The presence of the methylene signal at ∼3.7 ppm, corresponding to the −CH_2_– groups formed during the eugenol–eugenol condensation, further supports the formation of the biphenolic backbone.^35^

In the aromatic region (6.6–7.1 ppm), the proton resonance at ∼6.9 ppm, which in eugenol is associated with the aromatic position involved in the condensation pathway, shows a noticeable decrease relative to the other aromatic signals. Although absolute integrals are not shown, the reduction in the intensity of this resonance, together with the disappearance of the phenolic OH signal and the appearance of the new −CH_2_– linkage, is consistent with substitution at this aromatic site during the condensation step.^35,44^ These features confirm the successful condensation and epoxidation of eugenol into the target biobased monomer.

### 3.2 Curing Parameters and Kinetics for the Eugenol-Based Epoxy Resin

To facilitate a clearer understanding of how cross-linker chemistry influences the final properties of the materials, the BD formulation was included as a reference system. Although the BD network differs from BCA and BIA in both the nature of its interactions (epoxy-amine vs epoxy-carboxylic reactions) and the polymeric structure of the Jeffamine D230 hardener, it contains the same eugenol-based epoxy precursor and therefore provides a useful baseline. By comparing BD with carboxylic-acid-based systems, we can distinguish between the intrinsic contributions of the epoxy backbone and the specific effects introduced by carboxylic functionalities, such as increased acidity, hydrogen bonding, and susceptibility to hydrolysis. This approach allows us to highlight the unique behavior of carboxylated networks without implying a direct equivalence between the systems.

DSC analysis is a powerful technique for evaluating the curing parameters and kinetics of an ER-based system. The curing profiles of the synthesized epoxy networks and the corresponding reaction extents are presented in Figure 5a,b, while the extracted parameters are summarized in Table 2. The thermograms offer essential insights into the curing protocol required to obtain fully cross-linked thermosets. In this context, curing kinetics were determined at two different temperatures for each system, in accordance with the onset of the polymerization process and the temperature at which the curing rate reached its maximum (*T*_max_). Analysis of the evolution of the conversion curves describing the reaction between epoxy groups and the active functional groups (carboxylic or amine) from the curing agents reveals that most systems follow an auto-accelerated curing mechanism, evidenced by the steep initial slope of the conversion profiles.

**Figure 5:**
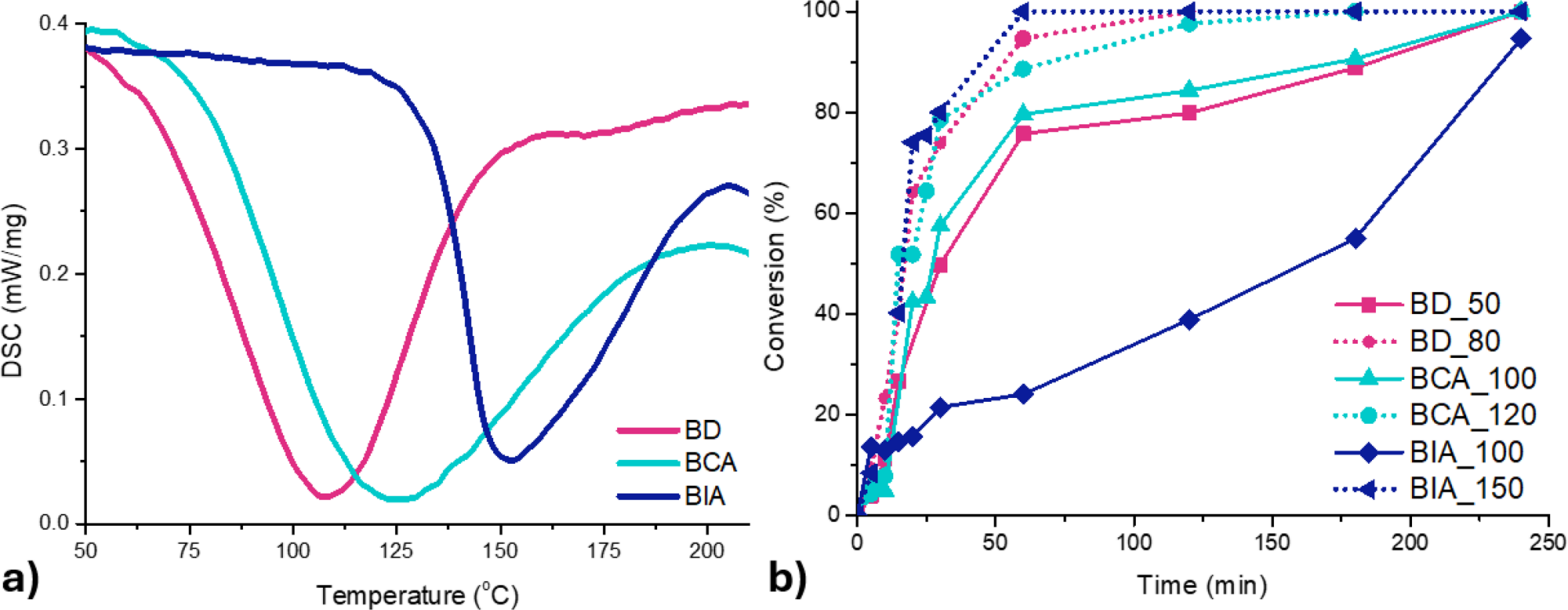
(a) DSC thermograms for biobased epoxy systems and (b) conversion of the curing reaction monitored by DSC for eugenol-based epoxy systems.

**Table 2:** Curing Data Extracted from the DSC Curves for Biobased Epoxy Systems

**sample**	**Δ*H*** (J/g)	***T*** **_max_** **(°C)**	**activation energy** (kJ/mol)
**BD**	192.9	107.9	81.8
**BCA**	235.4	123.4	25.8
**BIA**	128.2	152.6	45.5

However, the BIA system cured at 100 °C does not follow the same trend as the other formulations. This deviation is attributed to the specific structure and reactivity of itaconic acid, which contains both carboxylic groups and an α,β-unsaturated double bond. At lower temperatures, the epoxy-carboxylic acid esterification requires relatively high activation energy, and the rigid, hydrogen-bonded itaconate structure significantly limits molecular mobility. As a result, the BIA system exhibits low conversion at 100 °C, indicating that the reaction remains strongly diffusion-controlled under these conditions.

In contrast, curing at 150 °C provides sufficient thermal activation to overcome the kinetic barrier of the epoxy-acid reaction, reduce viscosity, and facilitate segmental mobility, allowing rapid progression of the cross-linking process. At this elevated temperature, the α,β-unsaturated double bond of itaconic acid may also participate in secondary reactions, such as thermal polymerization or radical-assisted cross-linking, further contributing to the higher conversion observed. The distinct features of the kinetic curves therefore reflect the combined influence of steric rigidity, hydrogen-bonding interactions, and dual functionality of itaconic acid, which give rise to a curing mechanism different from that of the amine- or citric-acid-based systems.

In the case of the BCA system, the high curing enthalpy indicates that a larger number of reactive sites are available for epoxy-amine cross-linking. However, the lower activation energy suggests that these groups are more easily accessible, less sterically hindered, or benefit from a local catalytic effect arising from the molecular structure of BCA. Notably, these two parameters are not directly correlated, and no monotonic trend between Δ*H* and Ea was observed. In this context, the higher curing enthalpy recorded for the CA-cured samples can be a consequence of secondary reactions, such as exothermic cyclization or inter- and intramolecular interactions. However, within the first 20 min of the polymerization, an induction stage is observed, which may correspond with the activation of the −COOH groups toward ester bond formation. After that, the process is accelerated and reaches a plateau, following the same trend independent of the thermal regime used.

The eugenol-based epoxy monomer cured with Jeffamine D230 exhibits a higher activation energy, which can be attributed to the long aliphatic structure of the primary amine cross-linking agent. However, the curing enthalpy indicates that a substantial number of chemical bonds are formed during the reaction. For the network cured at 80 °C, the maximum conversion was achieved after only 120 min, whereas the system cured at 50 °C reached full conversion after approximately 300 min. This pronounced difference highlights the strong influence of the temperature on the reaction rate during the autocatalytic stage. Moreover, the extended plateau between 80 and 100% conversion observed at 50 °C suggests that the final stage of curing proceeds through the diffusion-controlled mobility of the amino groups.

The curing behavior of the BD system differs substantially from that of the BCA and BIA formulations due to the structure of Jeffamine D230. The long polyether segment in Jeffamine imparts high chain flexibility and mobility, facilitating the epoxy-amine reaction and leading to faster curing kinetics and a lower cross-link density. In contrast, citric acid (BCA) and itaconic acid (BIA) are rigid, multifunctional small molecules, which promote more compact and highly cross-linked networks but require higher thermal activation. Therefore, the observed differences in enthalpy, activation energy, and conversion are closely related to the specific architecture and functionality of each curing agent. Comparisons among the systems are thus qualitative and aim to comparatively illustrate how the structural nature of the curing agents governs the curing pathway and the resulting network properties.

### 3.3 FTIR Analysis of Curing and Network Formation

FTIR analysis was performed on the eugenol-based epoxy networks both at the initial stage of curing and after full conversion to gain a better understanding of the structural modification that occurs during the cross-linking process.

As shown in figure 6, all samples display absorption peaks at 3484 and 3386 cm^–1^, corresponding to the stretching vibration of the hydroxyl group formed through the epoxy-ring-opening reaction. The intensity of this O–H stretching band increases considerably in the spectra collected after complete curing, further confirming the progression of the ring-opening mechanism. The high concentration of hydroxyl groups present in the fully cured networks is expected to enhance adhesion to various substrates. It is worth noting that the citric acid-cured sample exhibits only a slight difference between the initial and final spectra likely due to unreacted −COOH functionalities. This can be attributed to steric hindrance within the developing thermoset network, which limits full conversion of the carboxylic groups. Additionally, the absorption band in the 2966–2864 cm^–1^ region is associated with the −CH_2_ groups formed during epoxy-ring opening.

**Figure 6:**
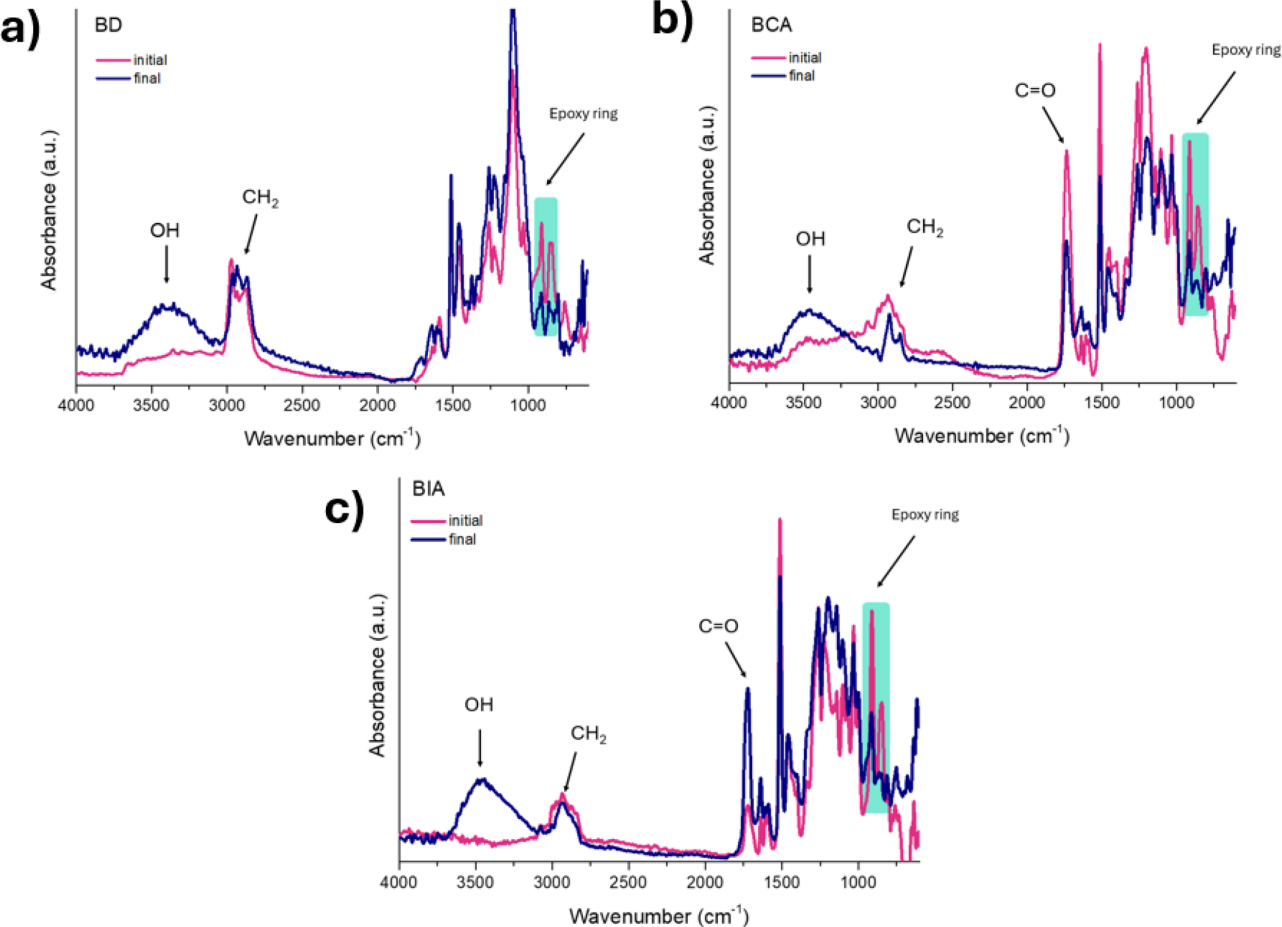
FTIR spectra of initial and final samples for (a) BD-, (b) BCA-, and (c) BIA-based epoxy formulations.

The progress of the curing reaction is further confirmed by the disappearance of the characteristic oxirane absorption bands at 928, 910, and 844 cm^–1^. These signals are present only in the spectra recorded at the beginning of the curing process, indicating the consumption of epoxy groups during network formation.^45^

In the case of BCA systems, in addition to the absorption peak at 1729 cm^–1^ corresponding to the C=O stretching vibration, several structural modifications can be observed. Specifically, the signal at 1030 cm^–1^ assigned to the ether C–O–C antisymmetric stretching shifts to 1110 cm^–1^. This shift confirms the presence of the C–O antisymmetric stretching characteristic band of ester groups and is consistent with a curing mechanism that proceeds through the reaction of the epoxy groups with the carboxylic acid functionalities.^38^

### 3.4 Thermogravimetric Analysis (TGA)

The thermal stability of the biobased epoxy network was evaluated through a TGA technique, and the corresponding results are presented in Figure 7. The influence of the curing agent on the cleavage mechanism of biobased networks is assessed by analyzing the benchmarks of thermal decomposition: onset degradation temperatures at 5 and 10% weight loss as an indicator for the thermal stability, the residual mass at 700 °C, and limiting oxygen index as guiding points toward flame-retardant coating materials.

**Figure 7:**
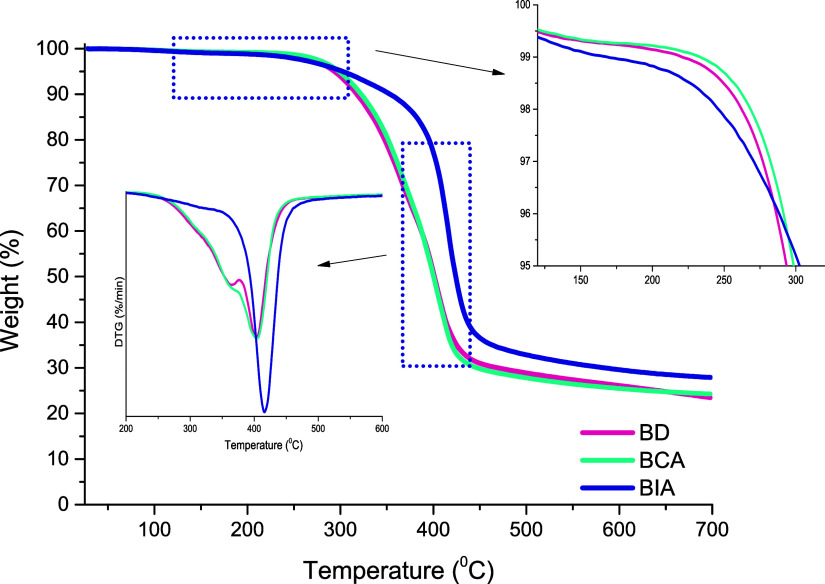
TGA curves of eugenol-based epoxy networks under a N_2_ atmosphere.

The thermal stability profiles of the cured epoxy networks (Figure 7) revealed no detectable weight loss below 100 °C, indicating the absence of a residual solvent. This confirms that the prepolymerization step effectively eliminated THF from the reaction mixtures.

The TGA curves of the biobased epoxy formulations exhibit three main degradation stages. The first stage, observed between 20 and 300 °C, corresponds to the initial thermal stability of the network. This is followed by the principal degradation step occurring between 300 and 450 °C, associated with the cleavage of labile bonds within the epoxy structure. The final stage, recorded between 450 and 700 °C, reflects the thermal oxidative stabilization of the remaining carbonaceous material, ultimately leading to char formation.

For the BCA-cured system, an additional decomposition event appears, attributed to the breakdown of the labile ester groups present in these networks. This process is evident as a shoulder in the derivative thermogravimetric (DTG) curve. In the case of the CA-cured samples, the first degradation step occurring between 200 and 260 °C corresponds to the degradation mechanism^46–48^ of ether and ester linkages, consistent with the expected thermal decomposition pathway.

The BIA systems presented the highest thermal stability and residual mass. The thermal degradation of this network proceeds through initial cleavage of ester linkages and subsequent oxidation of the aromatic backbone.^49^ In the BIA formulation, the dual functionality of itaconic acid creates a more compact, unsaturated network, which increases the thermal stability, shifts the main degradation peak to higher temperatures, and significantly enhances char formation. This enhanced performance is attributed to the participation of the α,β-unsaturated C=C bonds in secondary cross-linking reactions, generating a carbon-rich network with fewer readily degradable oxygenated moieties.

In contrast, the epoxy network cured with Jeffamine D230 exhibits a lower final thermal stability than the systems cured with carboxylic acids and yields the smallest residue amount. This outcome may result from incomplete consumption of the curing agent or from the accelerated degradation of residual poly­(ether chain)­s asymmetrically bonded to the epoxy monomer.

Another notable aspect is the increased char content observed for all of the samples. Conventional epoxy resins typically display values below 6%;^50^ accordingly, the 5-fold higher char yields obtained for the synthesized biobased epoxy networks strongly suggest enhanced flame-retardant properties. This characteristic makes the designed materials suitable for fire-resistant applications, as higher char formation can serve as a barrier to both volatile species generated during combustion and the underlying substrate, particularly in protective coating systems.

The limiting oxygen index (LOI) represents the minimum oxygen concentration (vol %) required to sustain combustion of a polymer after ignition and is considered a useful parameter for assessing flame-extinguishing ease. The LOI values (Table 3) calculated using the van Krevelen equation indicate that the eugenol-based networks exhibit self-extinguishing characteristics and show values comparable to conventional epoxy systems (DGEBA/DDM has an LOI of 26.2 ^51^). Moreover, the use of natural carboxylic curing agents appears to enhance the overall thermal behavior of the eugenol-derived networks. Notably, the highest LOI values observed for the BIA system may be associated with the network structure. The lowest molecular weight between cross-link points (Table 4) determined for this sample suggests a tightly arranged internal architecture within the bis-eugenol-based material, which can contribute to improved flame resistance.

**Table 3:** Thermostability and Char Content of Eugenol-Based Epoxy Systems

**sample**	***T*** **_d5%_** **(°C)**	***T*** **_d10%_** **(°C)**	***T*** **_max_** **(°C)**		**residual mass (%)**	**LOI**
**BD**	293.4	317.8	366.3	402.4	23.44	26.88
**BCA**	298.5	322.3	364.2	405.5	24.25	27.20
**BIA**	302.3	353.6	417.2		27.88	28.65

**Table 4:** Thermomechanical Characteristics for the Eugenol-Based Matrices

**sample**	**Tg (°C)**	**cross-linking density** **(mol/cm** ^**3)**^	**molecular weight between cross-linking points** (g/mol)	**GC (%)**
**BD**	55.2	5923	918.5	96%
**BCA**	55.9	1323	906.9	98%
**BIA**	47.8	5915	434.5	98%

### 3.5 Cured Network Properties

Thermomechanical properties of the synthesized biobased epoxy networks were evaluated through DMA, and the corresponding results are presented in Figure 8.

**Figure 8:**
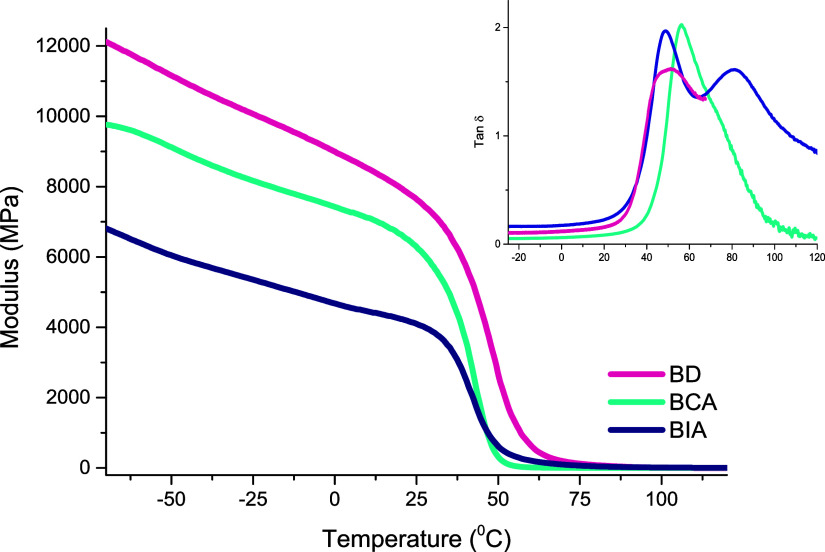
DMA curves for the cured bis-eugenol networks.

When the thermomechanical behavior is monitored through a bending test, the resulting curves (Figure 8) illustrate the stiffness of the networks in the glassy state between −50 and 75 °C. A comparison of the three systems indicates that the BD structure exhibits the highest stiffness likely due to the entanglement of the long polyether chains in Jeffamine D230, which contributes to a dense, flexible, yet tough network. In contrast, the lower storage modulus observed for the samples cured with carboxylic acids can be attributed to the chemical characteristics of the curing agents. Their short-chain nature may reduce segmental mobility and consequently limit chain entanglement. Furthermore, the carboxyl groups may prefer hydrogen-bonding interactions rather than forming strong covalent linkages, which can diminish the overall mechanical performance of the materials.

Analyzing the transition region between the glassy and rubbery state, it is observed that the BCA sample exhibits a relatively narrow glass transition domain, suggesting good network homogeneity. The shape of the tan δ curve (inset, Figure 8) further supports this interpretation. In contrast, the BIA network displays a broader and smoother transition, consistent with the bimodal profile of its tan δ curve. The temperature corresponding to the peak of each tan δ curve is defined as the Tg, and the values are summarized in Table 4. In general, the presence of two Tg peaks in DMA indicates distinct mobility restrictions for different chain segments, implying the formation of two types of microstructural domains within the epoxy network.^52,53^ This behavior may result from the double bond present in the IA structure, which can interfere with the curing reaction and introduce additional steric hindrance. The first peak (Tg = 47 °C) corresponds to more flexible chain segments, whereas the second peak (Tg = 82 °C) is associated with more constrained regions. The difference between the Tg of the carboxylic-acid-cured samples and the Jeffamine D230 system may be attributed to a plasticizing effect caused by the long, flexible amine chains.^50^

The similar Tg values for BD and BCA (∼55 °C) indicate that despite the distinct chemical architectures of their curing agents, both materials can form strong networks, either through flexible, entangled chains (BD) or through fewer but stiffer linkages (BCA). However, there are significant differences between the systems in terms of cross-linking density. The BD network exhibits the highest cross-linking density among the samples, consistent with the high reactivity of primary amine groups. In contrast, epoxy-carboxyl reactions proceed through esterification, which occurs at a slower rate than amino-epoxy reactions. Consequently, the rigid trifunctional carboxylic acid produces the lowest cross-linking density likely due to steric hindrance or secondary processes, such as intramolecular cyclization during thermal treatment. Notably, the itaconic acid-cured network displays a cross-linking density comparable to the BD system. This behavior may arise from the bifunctional nature of IA, which enables an efficient reaction with the epoxy ring. Complementarily, the cross-linking degree was assessed through gel content (GC) measurements (Table 4), representing the residual mass of cured materials after 24 h of immersion in THF. The results confirm the formation of dense networks with minimal unreacted monomer across all eugenol-based polymeric systems.

Despite its high gel content, the BCA-cured epoxy network exhibits a notably low cross-linking density (1323 mol/cm^3^), a behavior linked to steric hindrance and the limited reactivity of carboxylic groups. Although the formation of an extensive insoluble network confirms that curing was successful, the lower cross-link density indicates that fewer effective covalent bonds were created. Moreover, the high gel content in the BCA system may also arise from extensive hydrogen bonding among unreacted carboxylic and hydroxyl groups of citric acid and the surrounding epoxy matrix. These noncovalent interactions can reinforce network integrity and restrict molecular mobility, contributing to enhanced thermal stability and insolubility without increasing the measured cross-linking density.

The high gel content values (96–98%) confirm that all systems were fully cured; therefore, the relatively low glass transition temperatures arise mainly from the intrinsic flexibility of the eugenol-based backbone and the ester linkages introduced by the bioderived curing agents, rather than from incomplete cross-linking.

### 3.6 Surface Properties

Based on the most popular applications for epoxy resin as coatings and adhesives, the surface characteristics are key parameters that can predict the feasibility of these materials for advanced requirements in any of the previously mentioned areas.

The epoxy network cured with D230 exhibits a good hydrophilic behavior, reflected by its high surface free energy. Despite its flexible character, the relatively low contact angle compared with those of the BCA and BIA systems may also result from the participation of hydroxyl groups in physical interactions with the eugenol-based epoxy network, as previously discussed in the curing mechanism.

In contrast, the CA-cured sample displays a more hydrophobic behavior, as evidenced by its higher contact angle. This response may stem from the reduced number of hydroxyl groups available for surface interactions, originating either from unreacted citric acid moieties or from epoxy-ring opening during curing. The surface free energy value of 28.07 mN/m obtained for this system aligns with values reported in the literature for commercial epoxy resins.^54–56^ Furthermore, the differences in surface free energy between the BD and BCA systems can be attributed to the distinct chemical nature of their curing agents, which alters the polar component of the resulting networks. The notably lower surface free energy of the BIA system suggests an improved spreading behavior, making it potentially advantageous for coating applications.

Eugenol possesses intrinsic antibacterial properties, which can be efficiently leveraged in coating applications, particularly those exposed to moisture and environments that promote microbial growth. The antimicrobial activity of the eugenol-based thermosets was evaluated against *E. coli* strains, and the results are shown in Figure 9. A reduction in CFU values was observed across all coatings with the extent of antibacterial activity varying according to the curing agent used.

**Figure 9:**
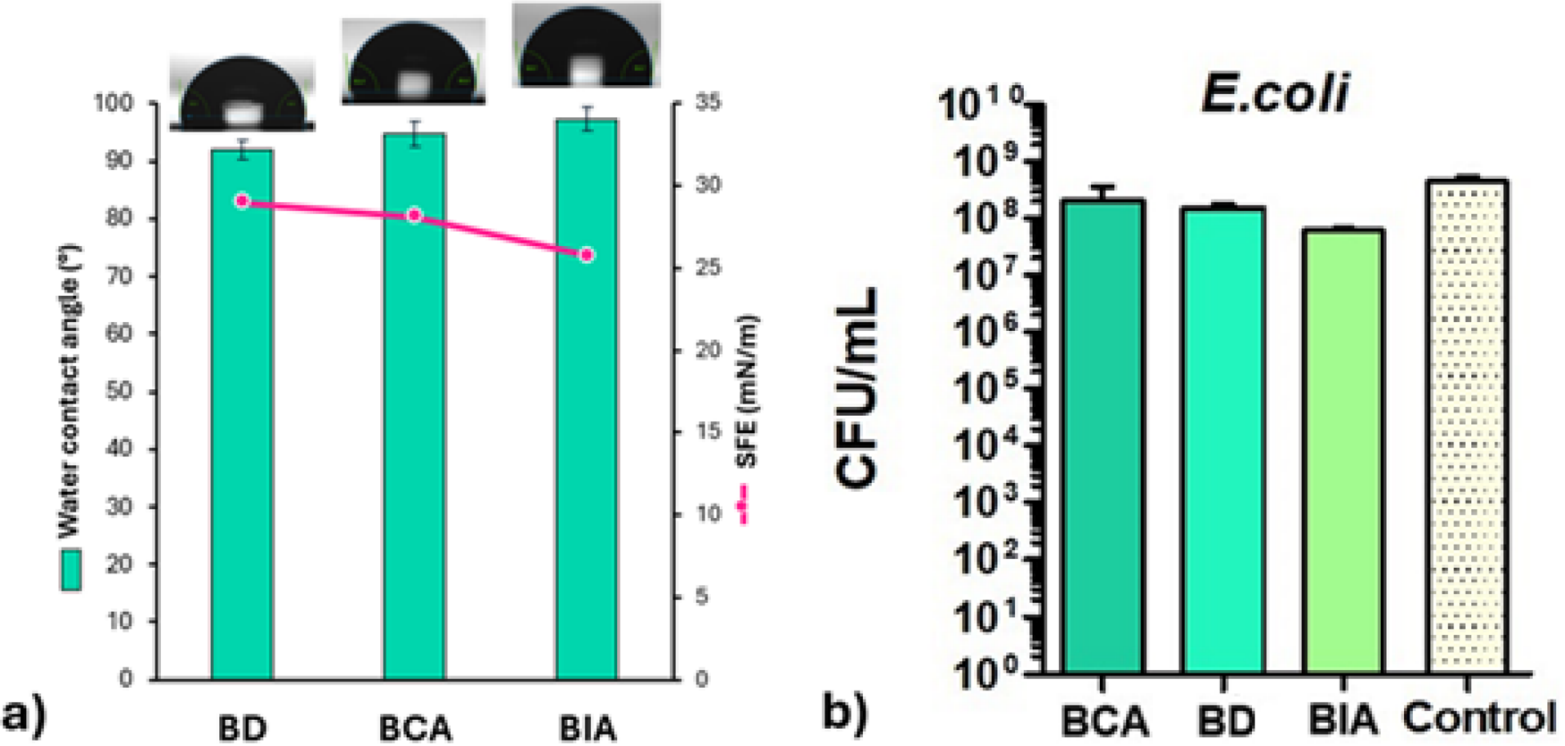
(a) Surface properties of the eugenol-biobased materials and (b) antibacterial features.

Carboxylic acids are widely known for^57,58^ their resistance to the most common bacterial strains. They have the ability to disrupt the membrane of the bacteria through different mechanisms, such as pH lowering and disturbance of microorganism’s metabolism. Therefore, in this study, BD was used as a comparative system without carboxylic groups, allowing the observation of the intrinsic behavior of the eugenol-based epoxy networks distinct from the specific contribution of carboxylic functionalities to antibacterial performance.

Thus, IA-cured thermoset exhibits the lowest affinity for the growth of *E. coli*. These findings are in agreement with the thermomechanical features of the BEF-IA network, confirming that the compact and entangled structure plays an important role in limiting the spreading of the bacteria onto the surface of the material.

Intriguingly, the overall antibacterial effect of the final coatings was not that significant, even though the components by themselves possess a high potential for such properties. A possible cause for this observation may be the involvement of the characteristic functional groups in the development of cross-linked structures, thus diminishing the bactericide effect.

### 3.7 Degradation Analysis

In the context of anticorrosive coating application, the resistance in different environment media is an important parameter. Figure 10 presents the behavior of eugenol-based systems under water, NaCl, H_2_SO_4_, and NaOH media at different immersion times.

**Figure 10:**
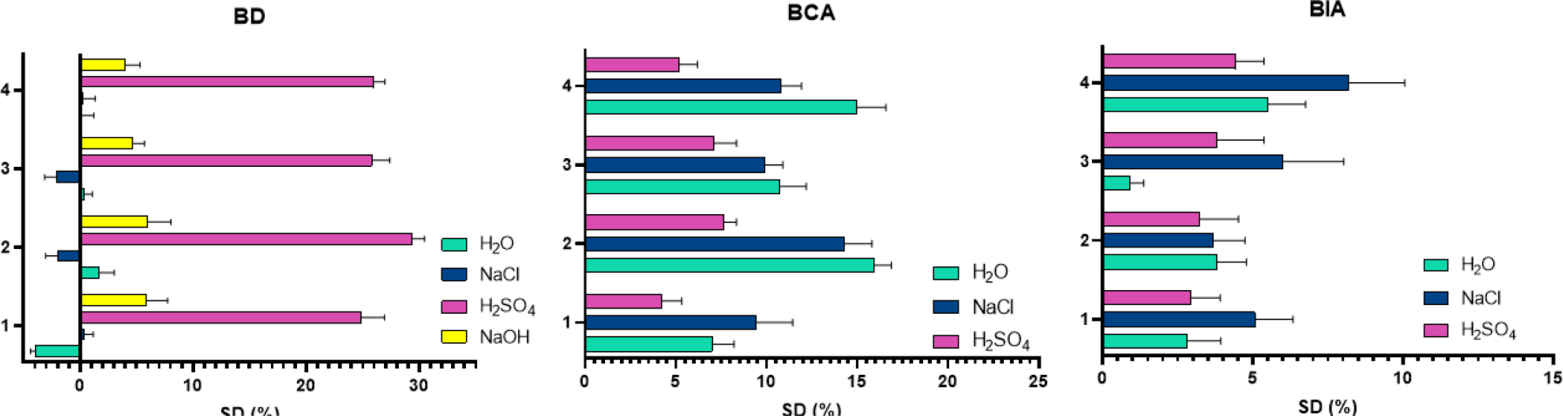
Degradation profiles for the eugenol epoxy network under different media measured at 1 day (1), 7 days (2), 10 days (3), and 14 days (4) of immersion.

As shown in the results, the BD material where the eugenol derivative is cured with conventional Jeffamine exhibits poor water stability, with degradation beginning on the first day of immersion. A similar trend is observed in saline solution, where deterioration becomes evident after 7 and 10 days, respectively. In contrast, the material demonstrates good resistance in both alkaline and acidic media; however, immersion in H_2_SO_4_ leads to pronounced swelling throughout the entire testing period. This response indicates not true resistance but rather a delayed corrosion process. Prolonged swelling of a protective layer typically promotes exfoliation, while the increased permeability of the swollen network facilitates the penetration of corrosive species, ultimately accelerating substrate degradation.

Eugenol-based materials cured with natural acids (CA and IA) show no resistance in alkaline media, undergoing decomposition within the first hours of exposure. In contrast, both the BCA and BIA systems exhibit high stability in acidic environments throughout the 14-day study, with the maximum SD increases of only about 5% after the full immersion period.

A comparison of the two eugenol-based materials cross-linked with naturally occurring curing agents (Figure 10) shows that the system containing the epoxy-itaconic acid linkage exhibits the best resistance to all studied corrosive media. This enhanced performance may arise from additional cross-linking reactions between the C=C double bonds of the eugenol-derived monomer and those of IA under the selected curing conditions. Such reactions can increase network rigidity and generate shorter segments between cross-linking points, as also indicated by the DMA-derived molecular weight between cross-links (∼435 g/mol), which is roughly half the value calculated for the BD and BCA systems (Table 4). Because of the reduced free volume within this more tightly packed network, penetration of solvents or aqueous media is significantly limited (maximum uptake of ∼7.5% in NaCl, ∼5% in water, and <5% in H_2_SO_4_). This restricted absorption minimizes swelling and prevents the degradation typically associated with excessive solvent diffusion, thereby contributing to the superior chemical resistance of the BIA material.

One of the main limitations of cured thermosets is related to the impossibility of recovering and reprocessing the polymeric network. Thus, by implying biobased cross-linkers, such as natural carboxylic acids, this drawback can be overcome. Due to the distinct chemical structure of the curing agents used for the development of the cross-linked network, it is important to assess the mechanism through which the epoxy backbone can be fragmented during the recycling process. The structural changes induced by the simulated corrosive media after degradation were assessed through XPS analysis, and the corresponding high-resolution C1s spectra are depicted in Figures 11–13. Beyond confirming that the extent of degradation varies with the cross-linker, XPS provides direct information about the chemical pathways involved, such as the cleavage of C–O bonds, the disappearance of ester groups, or the formation of oxidized species. These chemical changes at the surface allow us to distinguish whether degradation proceeds primarily via hydrolysis, oxidation, or disruption of unsaturated linkages.

**Figure 11:**
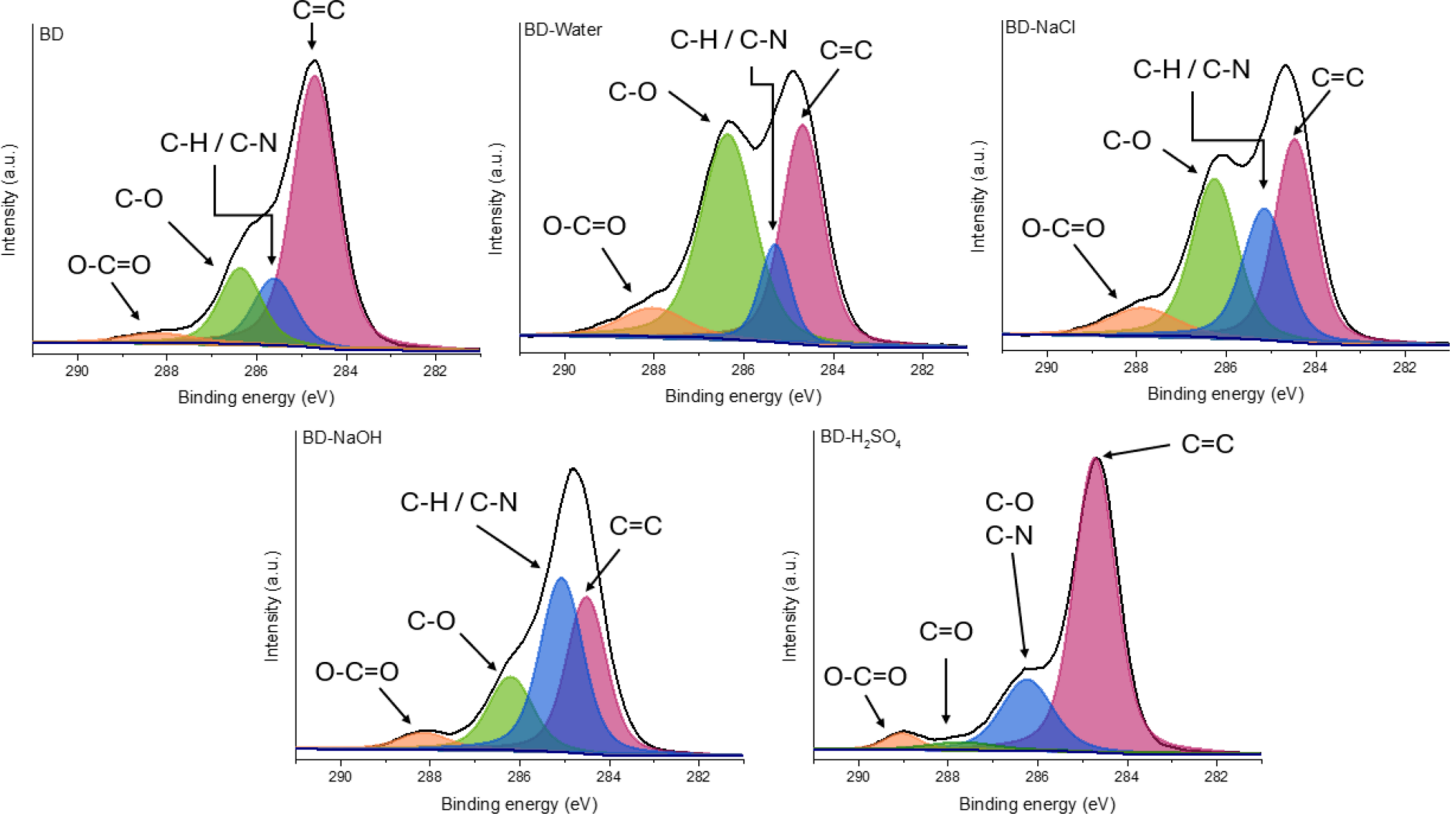
High-resolution C1s spectra for the BD samples after the swelling tests.

**Figure 12:**
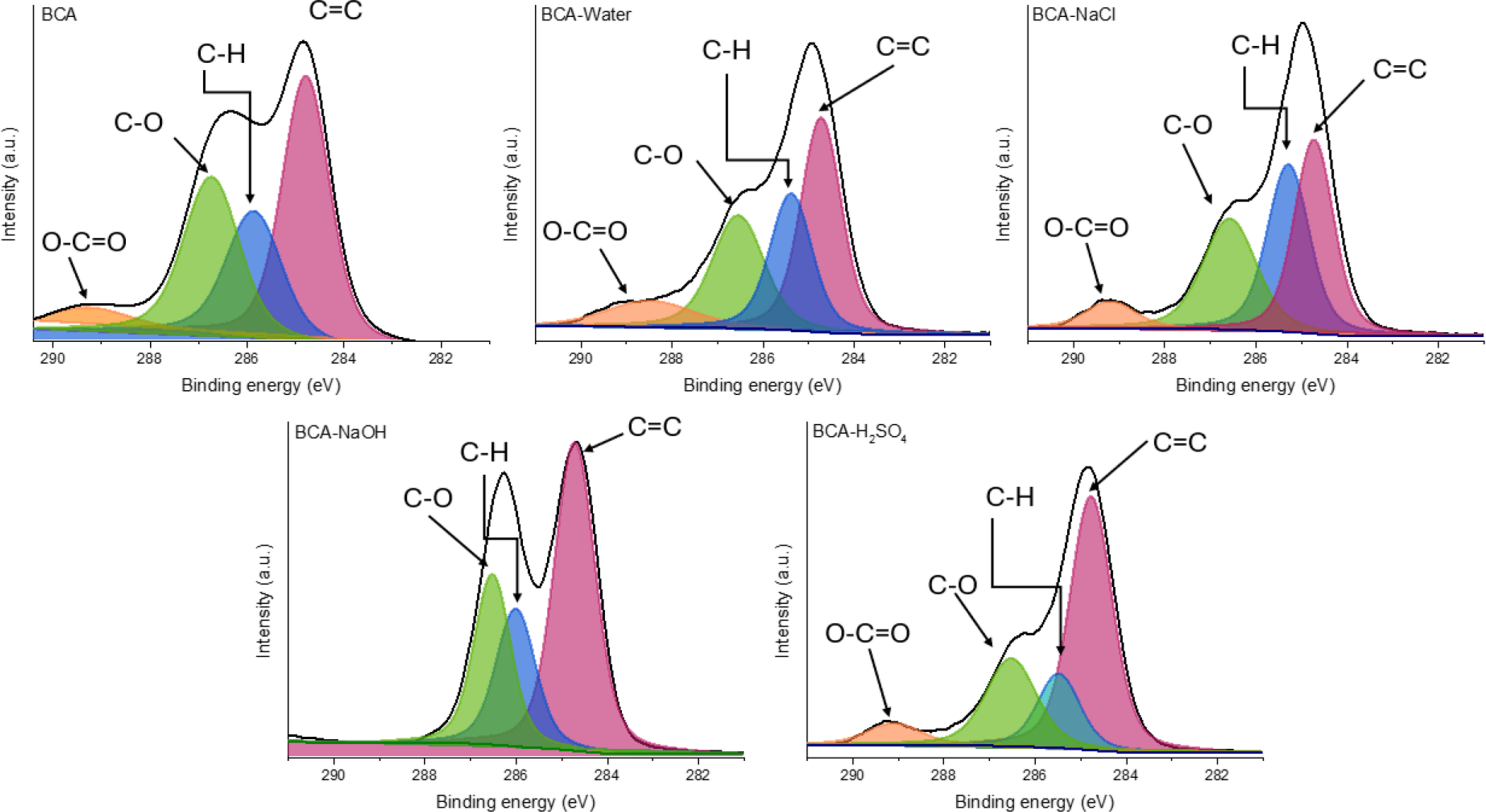
High-resolution C1s spectra for the BCA samples after the swelling tests.

**Figure 13:**
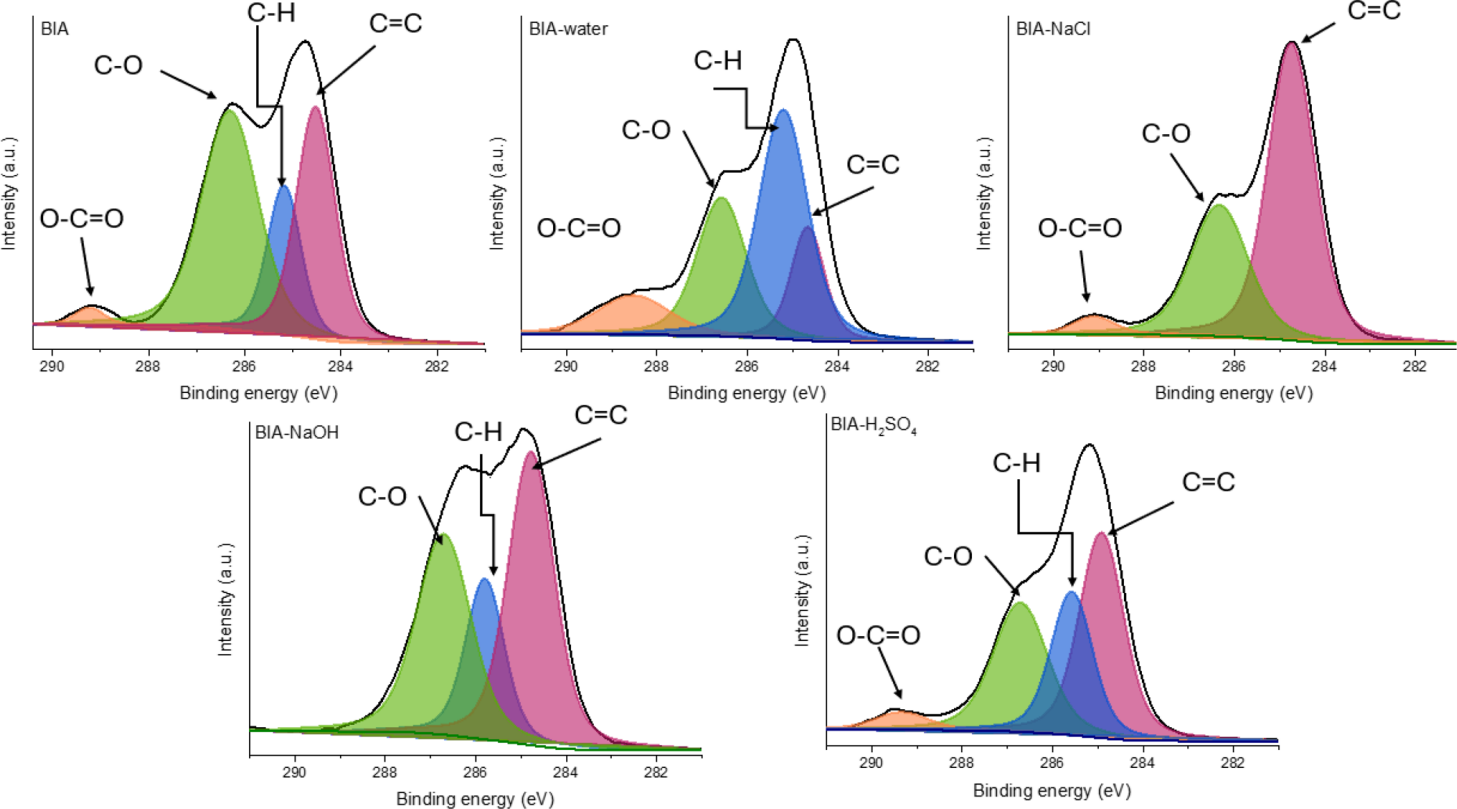
High-resolution C1s spectra for the BIA samples after the swelling tests.

The main signal from C *sp* ^*2*^ type located at 284.8 eV corresponds to the main polymeric backbone, the secondary peak from 285.2 eV is associated with C *sp* ^*3*^ species involved in C–H or C–N type bonding, mainly sourced by the cross-linker, and the third type of species observed at 286.3 eV are those coming from C–O type bonds of the epoxy network, The secondary peak located between 288 and 290 eV bears the particularity of each system.

The BD samples cured with synthetic polyether amine display four secondary signals for the C1s high-resolution spectra associated with the carbonaceous species highlighted in Figure 2. Upon exposure to aqueous media, cleavage predominantly occurs at the labile C–O segments of the network, revealing residual chains rich in terminal C–N groups. This effect is more pronounced in NaOH, as indicated by the increased intensity of the *Csp^3^* component in Figure 11, suggesting accelerated scission of ether linkages in alkaline conditions. In H_2_SO_4_, the oxidative environment converts C–O moieties into C=O and C–C=O species, further demonstrating that BD degradation proceeds mainly through oxidation or hydrolysis of oxygenated groups.

It is known that the ester bonds formed during the epoxy cure reaction with carboxylic acids are prone to hydrolysis. As shown in Figure 12, the surface structure of the citric-acid-cured samples is not significantly altered after exposure to aqueous solutions. These observations are consistent with the degradation profiles in Figure 10, which indicate swelling of the networks rather than immediate structural breakdown. However, the disappearance of the 289 eV signal corresponding to ester functional groups demonstrates that complete degradation occurs in NaOH, most likely through a β-hydroxy ester cleavage mechanism.^59^

Interestingly, the itaconic acid-cured matrices show a different conductivity, most likely due to a more complex mechanism by which the network is formed. This aspect was also evidenced through the more thermally stable material shown by TGA analysis, suggesting also double bond participation into network formation. When looking at the BIA samples immersed in water, the C *sp* ^*3*^ assigned peak is observed to be increased, probably due to a higher flexibility of the network upon swelling. Similarly, NaCl influences the C–O type species from the high-resolution C1s spectra exposed in Figure 13, along with the disappearance of the C–H-attributed secondary peak.

## 4 Conclusions

The effective development of eugenol-based bioepoxy networks cured with natural carboxylic acids and a traditional polyether amine is demonstrated in this work, emphasizing the significant impact of curing-agent chemistry on network formation and final material performance. The study revealed that the curing behavior and resulting cross-link architecture directly impacted the thermomechanical and surface characteristics of the systems. Notably, itaconic-acid-cured networks exhibited enhanced thermal stability, higher cross-link density, and improved flame-retardant potential with LOI values exceeding 28%, which are attributed to the rigid structure and additional cross-linking capability of the curing agent through both efficient epoxy-acid esterification and potential secondary cross-linking through the C=C bond.

On the other hand, the amine-cured systems showed larger flexibility, while the citric acid-cured systems showed lower cross-link density but characteristics similar to those of commercial epoxy coatings.

The BIA coatings displayed the lowest surface free energy, which maximizes the coating capability to spread over multiple substrates, and it is associated with better antibacterial activity against *E. coli*. The benefits of naturally occurring acid-cured systems were further highlighted by chemical-resistance experiments: BIA and BCA demonstrated strong stability in acidic media, limited swelling, and cross-linker chemistry-dependent degradation pathways, established by XPS. According to their structure, the ester-rich networks underwent hydrolysis in alkaline conditions. Overall, the results confirm that biobased curing agents provide an effective strategy for tailoring epoxy resin properties, enabling the design of sustainable materials with balanced performance suitable for coatings and related applications.

## Data Availability

All data supporting the findings of this study are available within the published article.
